# Impulsivity mediates the association between narcissism and substance-related problems beyond the degree of substance use: a longitudinal observational study

**DOI:** 10.1186/s12888-024-05718-y

**Published:** 2024-04-15

**Authors:** Malin K. Hildebrandt, Josepha Noack, Raoul Wuellhorst, Tanja Endrass, Emanuel Jauk

**Affiliations:** 1 Institute of Clinical Psychology and Psychotherapy, Chair of Addiction Research, Faculty of Psychology, Technische Universität Dresden, Chemnitzer Str. 46a, 01187 Dresden, Germany; 2 Department of Medical Psychology, Psychosomatics, and Psychotherapy, Medical University of Graz, Auenbruggerplatz 3, 8036 Graz, Austria; 3 Institute of Clinical Psychology and Psychotherapy, Chair of Clinical Psychology and Behavioral Neuroscience, Faculty of Psychology, Technische Universität Dresden, Chemnitzer Str. 46a, 01187 Dresden, Germany

**Keywords:** Narcissism, Addiction, Substance use disorder, Impulsivity, Substance use motives

## Abstract

**Background:**

Narcissism has been implied as a putative risk factor for substance use disorders (SUDs). However, previous research did not disentangle the degree of substance use from substance-related problems, the symptoms of SUDs. This preregistered study addressed the open question whether grandiose and vulnerable narcissism and their constituent traits convey specific SUD risk, that is, explain substance-related problems beyond the degree of use. Furthermore, we tested whether impulsivity or substance use motives linked to narcissistic self-regulation mediate this association.

**Methods:**

Narcissism, impulsivity, substance use motives, past-year substance use, and substance-related problems were assessed in 139 (poly-)substance users, 121 of whom completed a one-year follow-up. For significant longitudinal associations between narcissism factors and substance-related problems controlled for the degree of use, we tested impulsivity and substance use motives as mediators.

**Results:**

Grandiose narcissism (*r* =.24, *p* =.007) and its constituent factors antagonistic (*r* =.27, *p* =.003) and agentic narcissism (*r* =.18, *p* =.050), but not vulnerable narcissism, prospectively predicted substance-related problems beyond the degree of substance use. Associations of grandiose narcissism and antagonistic narcissism with substance-related problems were fully mediated by impulsivity, but not substance use motives. Impulsivity explained roughly one third of the association of both grandiose (P̂_M_ = 0.30) and antagonistic narcissism (P̂_M_ = 0.26) with substance-related problems.

**Discussion:**

We demonstrate that grandiose narcissism– particularly antagonistic but also agentic narcissism– is specifically linked to substance-related problems beyond the degree of substance use. The mediating effect of impulsivity but not substance use motives suggests that impulsivity may be a more important mechanism than narcissistic self-regulation in promoting SUD in narcissism. However, future studies may use more targeted measures than substance use motives to further probe the role of self-regulation. Similar result patterns for alcohol compared to all substances together indicate that mechanisms may be alike across substances. In conclusion, narcissistic individuals may not use substances more but have a higher SUD risk, informing prevention and treatment.

**Supplementary Information:**

The online version contains supplementary material available at 10.1186/s12888-024-05718-y.

## Background

Narcissism is characterized by a fragile sense of self and instable self-esteem [1, 2]. It has long been posited that individuals with narcissistic traits use psychoactive substances for self-regulatory purposes, suggesting this as a potential mechanism underlying the link between narcissism and substance use (cf. Jauk & Dieterich, 2019). Systematic research linked narcissism to substance use as well as to substance use disorders (SUDs; [3]; e.g. [4, 5]). However, much of this research (1) only focuses on selective substances (e.g., alcohol), (2) does not differentiate between substance use as compared to substance-related problems (the symptoms of SUDs), (3) is cross-sectional, and (4) does not speak to the putative mechanisms mediating the link of narcissism to SUDs. Therefore, the aim of this study was to investigate the longitudinal association of narcissistic traits and substance use as well as substance-related problems across different substances. Furthermore, we aimed to examine the mechanisms that underlie these associations by comparing impulsivity and substance use motives linked to narcissistic self-regulation as potential mediators.

### Dimensions of narcissism

#### The two-factor model: grandiose and vulnerable narcissism

Contemporary models differentiate grandiose and vulnerable narcissism. Both are characterized by self-importance and entitlement as core characteristics [6]. Beyond that common core, grandiose narcissism describes self-assured and dominant behavior [6] and vulnerable narcissism describes self-consciousness and withdrawal [7]. In the general population, grandiose and vulnerable narcissism are largely unrelated [6]. With increasing levels of grandiosity, however, grandiosity and vulnerability become more intertwined [8], consistent with pathological narcissism defined in terms of concurrent grandiosity *and* vulnerability [9]. Narcissistic personality disorder (NPD), as operationalized in the DSM, in contrast, is defined along extreme grandiosity [10], which can or cannot be accompanied by vulnerability [3].

#### The three-factor model: agentic, antagonistic and neurotic narcissism

**Fig. 1:**
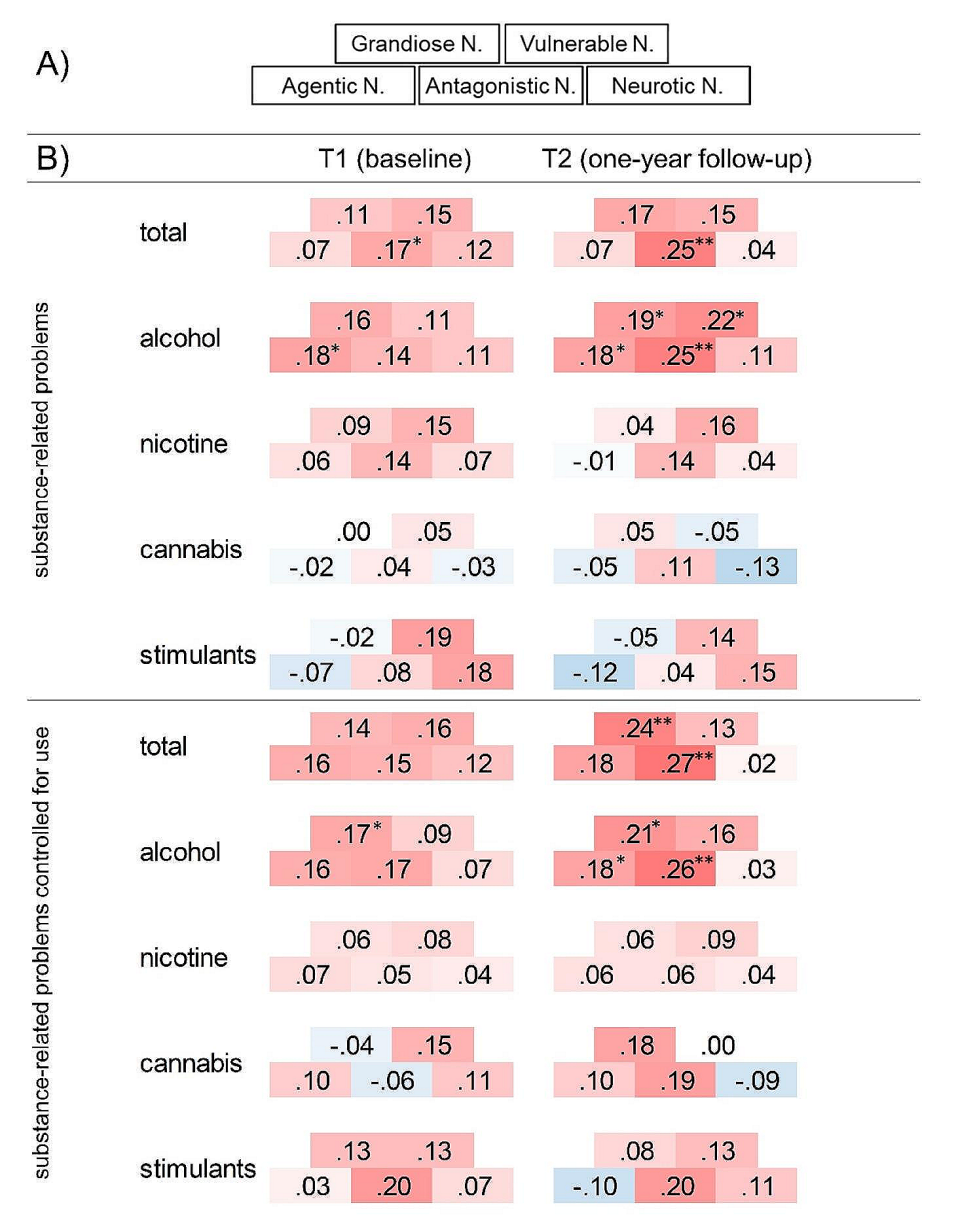
Correlations of narcissism dimensions with substance-related problems and partial correlations controlled for the degree of substance use. *Note*: (**A**) Schematic representation of the Trifurcated Model of Narcissism. (adapted from [70]) as a legend for (**B**) depicting heatmaps of cross-sectional and longitudinal correlations (upper panel) and partial correlations (lower panel; controlled for the degree of use) of the five dimensions of narcissism with substance-related problems. These are displayed for the total score (including all substances) as well as alcohol-, nicotine-, cannabis-, and stimulant-specific, respectively. Narcissism dimensions were assessed at T1. * *p* <.05, ** *p* <.01

While distinguishing grandiose and vulnerable narcissism has explained some paradoxical effects which have been associated with narcissism as a unitary construct, these broad factors still conflate aspects which might be related to different self-regulatory dynamics [11]. More fine-grained conceptualizations differentiate agentic, antagonistic, and neurotic narcissism [12, 13]. These reflect narcissistic variants of common Five-Factor Model (FFM) dimensions (e.g. [14]). In this model, grandiose narcissism can be described as a combination of antagonism (lower end of the agreeableness dimension, characterized by self-importance and entitlement) and agentic extraversion, while vulnerable narcissism can be described as a combination of antagonism and neuroticism, highlighting antagonism as the common core feature (see Fig. 1A). The FFM-based three-factor model of narcissism is an important extension as it often explains associations with external variables and their underlying self-regulatory mechanisms better [11].

### Narcissism and substance use

For associations between narcissism and substance use, studies either examined the presence of substance use (yes/no) or the degree of substance use (quantify/frequency of use). Grandiose narcissism was linked to a higher presence [15, 16] and degree of substance use (for a review see 4; [17–22]) This is contrasted by one study reporting that grandiose narcissism was associated with decreased smoking [23]. Comparable evidence for vulnerable narcissism is sparse and does not directly support an association with the degree of substance use [18, 22]. However, indirect evidence from studies on the foundational FFM traits in part supports a role of vulnerable narcissism in substance use. For the presence of substance use, a link to a combination of antagonism (low agreeableness) and neuroticism, the constituent traits of vulnerable narcissism, was reported [24, 25]. For the degree of substance use, there is contradictory evidence for [26] and against [25] an association with neuroticism, the FFM trait specific to vulnerable narcissism. Finally, studies assessing pathological narcissism in terms of concurrent grandiosity and vulnerability [9] also point to associations with the degree of substance use [27], although with very small effect sizes [22]. In sum, the evidence points towards an association of grandiose narcissism as well as, with weaker evidence, vulnerable narcissism with substance use. However, no study has examined associations of substance use with narcissism-specific measures of the three-factor model and some associations have only been reported with regard to alcohol use (e.g. of vulnerable traits with the degree of substance use [25, 26]), calling for a more fine-grained and comprehensive approach.

### Disentangling the degree of substance use and substance-related problems

SUDs are characterized by substance-related problems such as the inability to reduce or stop using the substance, or the failure to comply with social duties due to substance use. These substance-related problems are reflected in the symptoms of SUDs (DSM-5 A-criterion; [3]). Critically, only a fraction of frequent substance users develop substantial substance-related problems and thus SUDs (for instance, 22% for alcohol, 31% for cannabis, or 29% for cocaine; [28]). Thus, a high degree of substance use alone is neither sufficient nor necessary for an SUD diagnosis, rather, substance-related problems are crucial and distinguish substance-users with and without SUDs. In order to identify which factors are specific to this mental disorder, research should examine why only some substance users develop SUDs. This can be achieved by either comparing between substance users with and without SUD or, in a dimensional approach, computing associations with substance-related problems controlled for the degree of substance use [29, 30]. In contrast, past research has often relied on comparisons between SUD and healthy control groups. As these groups differ both in the degree of substance use and in substance-related problems, reported group differences may be attributable to differences in the degree of substance use rather than to a specific link to substance-related problems. The same holds for associations with substance-related problems when the degree of substance use is not (statistically) controlled for. Hildebrandt and colleagues recently showed that this was the case for sensation seeking, an often examined putative SUD risk factor [31]. The frequently reported association of sensation seeking with substance-related problems was explained by an underlying association with the degree of substance use. This demonstrates the necessity to disentangle associations with the degree of substance use and substance-related problems in SUD research.

### Narcissism and substance-related problems

Studies investigating the associations of narcissism and substance-related problems rarely disentangled the degree of substance use and substance-related problems and typically report categorical analyses based on SUD groups. For grandiose narcissism, the majority of the evidence relies on cross-sectional comorbidities between NPD and SUD [5, 15, 32, 33]. Furthermore, NPD longitudinally predicted the transition from being a non-user to being a “problematic user” (binary coding, minimum one substance-related problem; [34]), and an increased probability of having an SUD diagnosis [35]. In line with these categorical results, two dimensional studies report associations of grandiose narcissism with substance-related problems [10, 36]. However, none of these studies controlled for the degree of substance use as a potential confounder. In a study providing preliminary evidence on the specific link to substance-related problems, some aspects of grandiose narcissism were directly related to alcohol-related problems (entitlement rage) while others were only indirectly related (devaluing), mediated by an increased likelihood to engage in heavy episodic drinking (i.e. a high degree of substance use; [37]). This calls for examining the specific effect of three-factor model narcissistic traits on substance-related problems, ideally extending this preliminary evidence beyond alcohol use.

In other work, vulnerable (but not grandiose; [18, 37]) narcissism predicted substance-related problems [10] and relapse (indicating persisting substance-related problems), while grandiose narcissism was even a protective factor [38]. Furthermore, individuals with SUDs were characterized by vulnerable, but not grandiose narcissism (compared to healthy controls; [39]), and vulnerable narcissism was more strongly related to lifetime SUD than grandiose narcissism [40]. This is contrasted by reports of both higher grandiose and vulnerable narcissism in SUD [41] and a link between substance-related problems and pathological narcissism [42], characterized by co-occurring grandiose and vulnerable narcissism [9].

Almost all of the studies reported above rely on analyses that did not control for the degree of substance use, such that the reported associations with substance-related problems may be attributable to underlying associations with the degree of substance use. Only one study reports an analysis that controlled for the degree of substance use, namely an association of NPD (characterized by extreme grandiose narcissism, but clinically often accompanied by vulnerable narcissism) with nicotine dependence among current smokers [43]. This supports the notion that not only vulnerable but also grandiose narcissism may have an incremental relevance for substance-related problems that is not entirely explained by an underlying association of narcissism with the degree of substance use. However, these conflicting results highlight the necessity to move beyond the two-factor model and to control for the degree of substance use in the analyses to resolve these inconsistencies in the literature.

### Factors mediating the association between narcissism and substance use as well as substance-related problems

#### Impulsivity

Impulsivity is an umbrella term subsuming several interrelated traits [44] describing different aspects of the propensity to act quickly while disregarding long-term negative consequences [45]. Critically, impulsivity is related both to a high degree of substance use and to substance-related problems [45, 46]. We recently showed that urgency, an impulsivity-related trait describing the tendency to act rashly when experiencing (negative) emotions, explained incremental variance in substance-related problems when controlling for the degree of substance use [31]. Hence, impulsivity may contribute to a high degree of substance use, and, independently, to substance-related problems. In line with the special role of urgency for SUDs, it has been concluded that urgency may be a prime transdiagnostic endophenotype of mental health risk [47]. Consequently, we focused on urgency as our indicator of trait impulsivity in this study.

Impulsivity is discussed as a candidate mediator [48] because impulsivity is linked to narcissism (e.g. [49, 50]) and substance use as well as substance-related problems [31, 46]. Furthermore, antagonism, the core component of both grandiose and vulnerable narcissism, has been reported to mediate the association between impulsivity and substance-related problems [51]. However, the authors suggest that impulsivity may drive substance-related problems in narcissism through an antagonistic interpersonal style, implying that other factors than impulsivity alone may play a role.

#### Self-regulatory functions

A recent review proposed that other mechanisms than impulsivity may be more relevant for explaining associations between narcissism and substance use as well as substance-related problems [52]. Specifically, self-regulatory functions, for example affect regulation in self-relevant situations (contributing to the abovementioned interpersonal style), might be potential mechanisms of substance use in narcissism [17, 36, 52]. The three-factor model of narcissism (e.g. [12]), provides a framework for understanding affect regulation in narcissism. The central goal of agentic-narcissistic self-regulation is to maintain a grandiose self by means of self-enhancing strategies, whereas the central goal of antagonistic-narcissistic regulation is self-protection, for instance by means of aggressive behavior [53]. Neurotic narcissism, in contrast, is perceived as an exit strategy when the individual fails to maintain a grandiose self, and instead employs a “self-devaluation as self-protection” - strategy [53].

Narcissistic individuals may use substances as a self-regulation strategy serving these different goals. This should be reflected in different substance use motives or expectancies mediating the associations between different narcissistic traits and substance use as well as substance-related problems. A grandiose self-enhancement strategy should be reflected in motives of self-enhancement, such as increasing confidence through substance use. In contrast, a vulnerable “self-devaluation as self-protection” strategy may be reflected in coping motives, such as coping with resulting negative affect. Supporting these differential predictions regarding substance use motives for grandiose narcissism, a study showed that self-enhancement motives (to increase confidence), but not coping motives (to reduce tension) mediated the relationship of dark triad traits (including grandiose narcissism) with substance use [54]. Shame moderated the relationship between vulnerable narcissism and substance-related problems [36], indicating that coping with negative affect may play a role, and indirectly supporting our prediction regarding vulnerable narcissism. Further indirect evidence stems from research on the FFM traits underlying the three-factor model of narcissism. Extraversion (related to agentic narcissism) was linked to drinking to enhance, whereas neuroticism (related to neurotic narcissism) was linked to coping motives to drink ([55], for a review see [56]). Consequently, we expected that different substance use motives would play a role in the associations between grandiose vs. vulnerable narcissism with substance-related variables, and that substance use motives would explain these associations better than trait impulsivity. Our study is the first to test these two competing hypotheses, namely the impulsivity hypothesis versus the self-regulation hypothesis against each other. By using a more fine-grained model of narcissism, controlling for the degree of substance use to isolate specific effects on substance-related problems and providing longitudinal data, this study substantially adds to the preliminary and purely cross-sectional evidence in this field.

### Hypotheses

#### Confirmatory hypotheses (preregistered)

These hypotheses were preregistered in the Open Science Framework (https://osf.io/r2cmp; 23rd of December 2021). We expected that grandiose as well as vulnerable narcissism would be associated with the degree of substance use as well as with substance-related problems. For grandiose narcissism, we expected that these associations would be mediated by impulsivity and enhancement motives, with enhancement motives being the stronger mediator. For vulnerable narcissism, we expected that these associations would be mediated by impulsivity and coping motives, with coping motives being the stronger mediator. Although an indirect (i.e. mediation) effect may be present in the absence of a total effect (i.e. association the mediation is tested upon; [57]), to avoid false positives facilitated by a large number of tests, we planned to conduct the respective mediation analyses only if the underlying association was significant. We based the mediation analyses on one-year follow up data on substance-related variables in order to highlight the temporal stability of the effects. Given the sparse literature, we had no differential hypotheses between substances, such that we tested all hypotheses separately for a total measure of all substances as well as the substances alcohol, nicotine, cannabis, and stimulants.

#### Exploratory hypotheses

In addition to the preregistered hypotheses, we also investigated whether dimensions of narcissism explained incremental variance in substance-related problems beyond the degree of substance use. Furthermore, to illuminate which constituent dimensions were driving the effects in grandiose and vulnerable narcissism, we explored the associations of dimensions of narcissism and substance use outcomes within the three-factor model (i.e., we sought to clarify whether effects for grandiose narcissism are more due to agentic or antagonistic aspects, and effects of vulnerable narcissism are more due to antagonistic or neurotic aspects).

## Method

### Participants

We recruited participants mainly from the general population through advertisements and flyers in clubs, bars, and counselling centers, and through postings on websites associated with the electronic music scene, al as well as from a precursory study [31]. The data used in the present study represent a subsection of a larger project which is available at https://osf.io/cwnrg/.

Inclusion criteria were (1) current use of at least one substance once per month (2), age between 18 and 35 years (3), native German speakers or learned before the age of 10 years (4), right-handed (5), first substance use at least one year ago (6), no report of withdrawal symptoms in periods when participants used substances to a similar degree as in the past three months (7), no reported use of any substance (except for nicotine) for at least the five-fold of the respective plasma half-life prior to testing [58], (8) current and previous neurological and psychological health status according to MRI guidelines from the university’s neuroimaging center, and (9) physical demands like ability to move the fingers, normal or corrected-to-normal vision, no cardiovascular disease, no pregnancy, no nursing infants nor implants contraindicated in MRI.

The present study reports results based on those participants from the larger project who provided complete data on all necessary variables. Out of the 142 participants who came to the laboratory, two did not complete the assessment and one was excluded for a current medical condition that may have affected the data, yielding a final sample of *N* = 139 participants (T1), 122 of whom completed a one-year follow-up assessment including repeated measures of substance use and substance-related problems (T2). We conducted post-hoc power calculations based on effect size estimates stemming from the only study reporting mediation analyses resembling our preregistered hypotheses, focusing on the weaker of both eligible mediation effects to yield conservative estimates (tension reduction, an indicator of coping motives, as a mediator; [54]). A power analysis based on the Sobel test determining significance of a mediation effect [59] given a power of 0.8 and a two-tailed 𝛼 of 0.05 indicated that a sample of 119 participants would be needed. Furthermore, the bias-corrected bootstrapping approach we applied in this study requires slightly smaller sample sizes than the Sobel test to uncover a true mediation effect [60]. Hence, our sample size should be sufficient for the models including all participants.

Table 1 presents sociodemographic characteristics of the sample. Participants predominantly self-identified as white (see supplemental Table S1) and received 50€ at T1 and 20€ at T2 or course credit (*n* = 2). The study followed the guidelines stated by the Declaration of Helsinki [61].

**Table 1:** Sociodemographic and substance use characteristics

Characteristic	Laboratory session		Follow-up	
	N	%	N	%
Gender (f/m/d)	54/82/3	39/59/2	49/72/2	40/59/1
	M	SD	M	SD
Age	24.8	4.5	25.7	4.3
Degree of substance use	110.1	76.4	94.7	68.1
Substance-related problems	13.1	11.7	11.4	10.9

*Note*: *M* and *SD* represent mean and standard deviation, respectively

### Procedure

In the laboratory session (T1), participants completed behavioral and functional MRI-paradigms as well as questionnaire measures and a structured clinical interview including all measures relevant to this study. The one-year follow-up (T2) was completed 12 to 15 months later and included repeated assessments of the degree of substance use and substance-related problems. The OSF project page provides further detail on the study procedures (https://osf.io/jqc3d).

### Materials

#### Narcissism

**Brief form of the Five-Factor Narcissism Inventory (FFNI-BF)**. The brief form of the Five-Factor Narcissism Inventory (FFNI-BF; 11) consists of 30 items (five-point Likert scale ranging from 1 to 5 reflecting “disagree strongly” to “agree strongly”). Sum scores describe grandiose (22 items) and vulnerable narcissism (8 items; two-factor model) as well as their constituent dimensions agentic narcissism (8 items), antagonistic narcissism (16 items), and neurotic narcissism (6 items; three-factor model). Higher scores indicate stronger expression of the respective traits. The FFNI-BF is based upon the English 148-item FFNI [62] and was found to have similar reliability and in some aspects even advantageous validity [11].

#### Degree of substance use and substance-related problems

**Degree of substance use: Dresden Inventory of Substance Use (D-ISU).** This questionnaire is designed to assess lifetime as well as current substance use for each individual substance ever used by a participant [29]. Beyond lifetime measures not relevant for this study, for each substance used in the past 12 months, participants indicated use frequency (number of use occasions), subjective quantity of use (six-point Likert scale ranging from 0 to 5 reflecting “nothing” to “very much”), as well as objective quantity of use (e.g. cigarettes, grams) on a typical occasion within the past 12 months. For each substance, the product of frequency (use occasions) and subjective quantity forms the *substance-specific degree of substance use*, an approximation of cumulative use quantity over the past year. The use of subjective quantity scores for the computation of the *substance-specific degree of substance use* scores, necessary to allow accumulating across substances, was validated by strong correlations between subjective and objective quantity scores [29]. The *total degree of substance use* is the sum of all *substance-specific degree of substance use* scores.

To limit the number of comparisons while allowing to examine substance-specific results, we analyzed the four most commonly used substances, namely *alcohol*, *nicotine*, *cannabis*, and *stimulants* (composite of amphetamine, methamphetamine, and cocaine), as well as the *total degree of substance use* comprising all substances used by a participant (not limited to alcohol, nicotine, cannabis, and stimulants), yielding five degree of substance use variables.

**Substance-related problems: Structured Clinical Interview for Psychological Disorders (SCID-5 CV).** Trained interviewers conducted the Structured Clinical Interview for DSM-5 Disorders Clinician Version (SCID-5 CV, [63]) which assesses psychological disorders, including SUD, based on the DSM-5 criteria [3]. We adapted the SCID-5 CV for this study by excluding subsections referring to diagnoses that had already been screened during the telephone interview, and introducing a severity coding for SUD symptoms (four-point Likert scale ranging from 0 (“not at all”) to 3 (“extreme”)) to obtain greater variance in the substance-related problems measure. The SUD subsection of the interview was conducted for every substance that a participant had used more than 5 times in the past 12 months and refers to the time period of the past 12 months. For each substance, we computed the sum of all symptom severity ratings yielding substance-specific substance-related problems. The total score of substance-related problems is the sum of all criterion severity ratings for all substances.

#### Impulsivity

**UPPS-P Impulsive Behavior Scale (UPPS-P).** Impulsive traits were assessed using the UPPS-P Impulsive Behavior Scale [64], German version, which consists of 59 items (four-point Likert scale ranging from 1 to 4 reflecting “agree strongly” to “disagree strongly”) and has shown good psychometric properties [65]. Besides other impulsivity-related traits not relevant for this study, the UPPS-P assesses *negative urgency* (sum of 12 items, higher scores represent stronger expression), the tendency to act rashly when experiencing negative emotions, which we used to operationalize trait impulsivity.

#### Self-regulation

**Enhancement and Coping Motives**: **Substance Use Motives Measure (SUMM).** The Substance Use Motives Measure [66] identifies eight motives for substance use using 32 items (five-point Likert scale ranging from 1 to 5 reflecting “not at all” to “very frequently”). We were interested in the SUMM subscales related to enhancement and coping with negative affect, each reflecting the mean of 4 items, namely *enhancement*, *anxiety-coping*, and *depression-coping with higher scores representing stronger expression of the respective motive.* As we did not have differential predictions regarding coping with anxiety as compared to depression, we computed the sum of these subscales to form the variable *coping motives*, in order to limit the number of comparisons.

The SUMM assesses use motives for one specific substance. Due to time restraints, participants did not fill out the SUMM for every substance they used but only for the two substances they reported as the currently most relevant ones. We computed total coping motives and total enhancement motives as the sum of the two substance-specific scores for each participant. The SUMM has shown good internal consistency and convergent validity [66]. The SUMM is not available in German and was therefore translated into German and back into English by two independent individuals within the scope of the larger project.

### Data preprocessing and statistical analyses

All analyses were conducted in R [67]. Regarding specific substances, our substance use data convey two different kinds of information, which we analyzed separately. First, to examine whether substance users and non-users of a specific substance differed in narcissism, we created a binary variable indicating use or no use in the past 12 months. Second, to examine associations with the degree of substance use, we created substance-specific subsamples including only those individuals who had used the respective substance in the past 12 months to address zero-inflation. We transformed variables with skewed distributions with the ordered quantile normalization transformation [68], either within the substance-specific subsamples (for substance-specific analyses), or within the full sample.

To identify whether narcissism was associated with substance use, we compared the mean expression of each narcissism dimension between substance users and non-users for each substance using independent sample t-tests. To identify whether narcissism was associated with the degree of substance use as well as substance-related problems, we computed bivariate Pearson’s correlations between each of the narcissism dimensions (two-factor model: grandiose narcissism and vulnerable narcissism, three-factor model: agentic narcissism, antagonistic narcissism, neurotic narcissism) and the degree of substance use as well as substance-related problems, for the total scores and each substance separately. To examine whether narcissism dimensions predicted substance-related problems beyond the degree of substance use, we computed partial correlations between narcissistic traits and substance-related problems controlled for the degree of use, for each narcissism dimension and for the total scores as well as each substance separately.

We conducted mediation analyses only when the underlying associations were statistically significant. We used the PROCESS-macro for R, an ordinary least squares and logistic regression path analysis modeling tool [69], model 4.2 for the parallel mediation analyses, and model 6 for the sequential mediation analysis, respectively. Each mediation analysis included the degree of substance use or substance-related problems as dependent variable, the respective factor of narcissism as an independent variable, and the mediator variables (1) coping motives (for vulnerable antagonistic and neurotic narcissism) and/or enhancement motives (for grandiose, antagonistic and agentic narcissism) and (2) impulsivity (negative urgency). The models predicting substance-related problems additionally included the degree of substance use as a control variable. Given a significant indirect effect of a substance use motive, we computed the difference between the indirect effect mediated by this mediator and the indirect effect mediated by impulsivity and used a bootstrapped confidence interval to determine if substance use motives were stronger mediators than impulsivity. The analyses testing the mediation hypotheses were based on subsamples consisting of participants who provided SUMM data for the respective substance.

## Results

### Associations of narcissism with the degree of substance use and substance-related problems

#### Degree of substance use

Across all substances, substance users did not differ significantly from non-users in any narcissism dimension (all *p*s > 0.05, see supplemental Table S2), validating the use of substance-specific subsamples to examine associations with the degree of substance use. Against expectations, no narcissism dimension was significantly correlated with total or substance-specific degree of substance use scores (all *p*s > 0.05, see supplemental Table S3).

#### Substance-related problems

Narcissism dimensions showed consistent patterns of association with substance-related problems across both measurement points with stronger prospective than cross-sectional effects. For bivariate associations of narcissism dimensions with substance-related problems (not controlling for the degree of substance use), the effect was strongest for alcohol, but also reflected in total substance-related problems. In line with the preregistered hypotheses regarding the two-factor model of narcissism, both grandiose (*r* =.19, 95% CI [0.01, 0.36], *p* =.034), and vulnerable narcissism (*r* =.22, 95% CI [0.05, 0.39], *p =*.013) prospectively predicted alcohol-related problems at follow-up. These effects had the same direction in the cross-sectional data on alcohol as well as for total substance-related problems but were not significant (all *p*s > 0.05, see Fig. 1). Examining the heatmap of correlations (Fig. 1B, upper panel; for confidence intervals see supplemental Table S3) reveals that these associations of vulnerable and grandiose narcissism with substance-related problems seem to be most strongly driven by their common constituent trait antagonistic narcissism, which overall shows the strongest associations with substance-related problems, even more pronounced longitudinally (total: *r* =.25, 95% CI [0.07, 0.41], *p* =.006; alcohol: *r* =.25, 95% CI [0.08, 0.41], *p* =.005). Furthermore, only for alcohol, agentic narcissism was also significantly associated with alcohol-related problems, both cross-sectionally (*r* =.18, 95% CI [0.01, 0.34], *p* =.034) and longitudinally (*r* =.18, 95% CI [0.00, 0.35], *p* =.049). For nicotine-, cannabis- and stimulant-related problems, there were no significant associations with narcissism dimensions.

#### Substance-related problems controlled for the degree of use

Bivariate correlations of narcissism dimensions with substance-related problems may be explained by underlying differences in the degree of substance use, which we addressed by statistically controlling for the degree of substance use in this exploratory analysis. Examining the heatmap of partial correlations (Fig. 1B, lower panel; for confidence intervals see supplemental Table S3) suggests that this was not the case. Rather, the association of antagonistic narcissism with substance-related problems seems be specific to the development of substance-related problems, and thus SUDs. This is reflected in pronounced longitudinal associations with total (*r* =.27, 95% CI [0.09, 0.42], *p* =.003) and alcohol-specific substance-related problems (*r* =.26, 95% CI [0.08, 0.42], *p* =.004), and further supported by associations with cannabis- (*r* =.19, 95% CI [-0.02, 0.38], *p* =.077) and stimulant-related problems (*r* =.20, 95% CI [-0.03, 0.41], *p* =.097). Note that the latter of these correlations were not significant at conventional thresholds, potentially due to the smaller sample size of the subsamples, yet displayed similar effect sizes (see Fig. 1B). The longitudinal specific associations of antagonistic narcissism with total and alcohol-related problems beyond the degree of use were also reflected in the superordinate factor grandiose narcissism (total: *r* =.24, 95% CI [0.07, 0.40], *p* =.007, alcohol: *r* =.21, 95% CI [0.03, 0.37], *p =*.021). Last, agentic narcissism prospectively predicted alcohol-related problems beyond the degree of substance use (trend level for total substance-related problems: *r* =.18, 95% CI [0.00, 0.34], *p* =.050, alcohol: *r* =.18, 95% CI [0.00, 0.35], *p* =.047).

### Are associations of narcissism with substance-related problems mediated by impulsivity and substance use motives?

We conducted mediation analyses for the five significant longitudinal associations between narcissistic traits and substance-related problems controlled for the degree of substance use.

#### Confirmatory mediation analyses based on the two-factor model

**Fig. 2:**
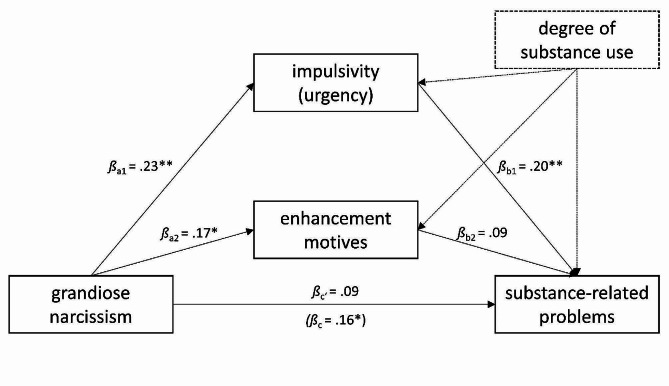
Parallel mediation model linking grandiose narcissism to substance-related problems controlling for the degree of substance use. *Note*: *N* = 123. Substance-related problems were assessed at T2, all other variables at T1. * *p* <.05, ** *p* <.01

**Mediation of the association between grandiose narcissism and substance-related problems**. The regression of substance-related problems on grandiose narcissism, controlling for the degree of substance use, was significant (*β*_c_ = 0.16, *p* =.030). Grandiose narcissism significantly predicted impulsivity (*β*_a1_ = 0.23, *p* =.008), and impulsivity subsequently predicted alcohol-related problems (*β*_b1_ = 0.20, *p* =.008). In contrast, grandiose narcissism predicted enhancement motives (*β*_a2_ = 0.17, *p* =.048), but enhancement motives did not subsequently predict alcohol-related problems (*p* >.05). Consistently, the indirect effect through impulsivity was significant (*β*_a1b1_ = 0.05, 95% CI [0.003, 0.114]) while the effect of grandiose narcissism on substance-related problems was no longer significant, indicating a complete mediation (see Fig. 2). Approximately one third of the total effect was explained by the indirect effect (P̂_M_ = 0.30; [71]).

**Fig. 3:**
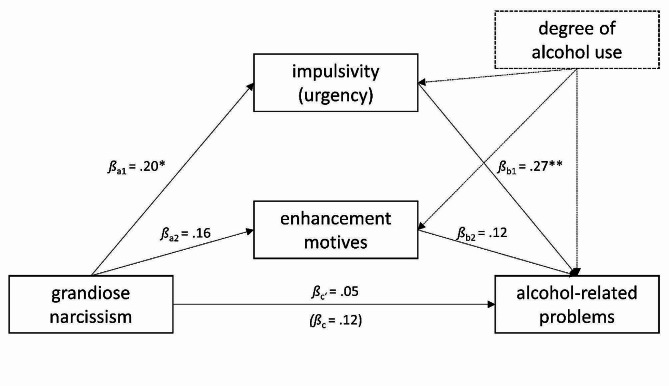
Parallel mediation model linking grandiose narcissism to alcohol-related problems while controlling for the degree of alcohol use. *Note*: *N* = 98. Alcohol-related problems were assessed at T2, all other variables at T1. * *p* <.05, ** *p* <.01

**Mediation of the association between grandiose narcissism and alcohol-related problems**. The regression of alcohol-related problems on grandiose narcissism, controlling for the degree of alcohol use, was not significant (*β*_c_ = 0.12, *p* =.19). Note that this is not equivalent to the partial correlation reported above, as the mediation model relies on the subsample that provided SUMM data concerning alcohol (*N* = 98) and thus has less power. Grandiose narcissism significantly predicted impulsivity (*β*_a1_ = 0.20, *p* =.049), and impulsivity was subsequently related to alcohol-related problems (*β*_b1_ = 0.27, *p* =.004). In contrast, grandiose narcissism was unrelated to enhancement motives and enhancement motives were not subsequently related to alcohol-related problems (all *p*s > 0.05). The indirect effect through impulsivity was not significant (*β*_a1b1_ = 0.05, 95% CI [-0.011, 0.153], see Fig. 3).

Importantly, the effect size of the indirect effect of impulsivity was similar to the effect size in the model predicting total substance-related problems (see “Mediation of the association between grandiose narcissism and substance-related problems”), such that the failure to find a significant effect here may be attributable to the different sample sizes implying attenuated power. Supporting this interpretation, approximately half of the total effect was explained by the indirect effect (P̂_M_ = 0.44; [71]).

Taken together, the association of grandiose narcissism with substance-related problems may be mediated by impulsivity and similar patterns of associations based on total substance-related problems and alcohol-related problems suggest that this may be similar across substances.

#### Exploratory mediation analyses based on the three-factor model

On an exploratory basis, we further examined which variables mediated the associations of antagonistic and agentic narcissism, the constituent factors of grandiose narcissism, with substance-related problems beyond the degree of substance use. This study is based on the assumption that enhancement motives may result from a grandiose self-regulation strategy and coping motives may result from a vulnerable self-regulation strategy. Consequently, we tested both motives as mediators in the models including antagonistic narcicssism (as the common core of grandiose and vulnerable narcissism), and only enhancement motives as a mediator in models including agentic narcissism (as the uniquely grandiose factor).

**Fig. 4:**
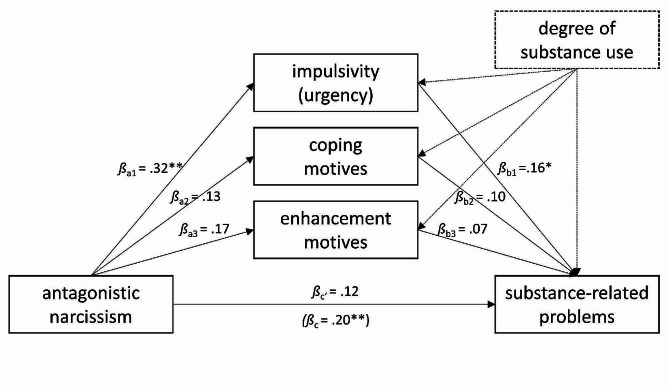
Parallel mediation model linking antagonistic narcissism to substance-related problems while controlling for the degree of substance use. *Note*: *N* = 123. Substance-related problems were assessed at T2, all other variables at T1. * *p* <.05, ** *p* <.01

**Mediators of the association between antagonistic narcissism and substance-related problems**. The regression of substance-related problems on antagonistic narcissism, controlling for the degree of substance use, was significant (*β*_c_ = 0.20, *p* =.007). Antagonistic narcissism significantly predicted impulsivity (*β*_a1_ = 0.32, *p* =.000), and impulsivity subsequently predicted substance-related problems (*β*_b1_ = 0.16, *p* =.039). In contrast, antagonistic narcissism did not significantly predict enhancement or coping motives and neither enhancement nor coping motives did subsequently predict substance-related problems (*p* >.05). Consistently, the indirect effect through impulsivity was significant (*β*_a1b1_ = 0.05, 95% CI [0.003, 0.124]) while the effect of antagonistic narcissism on substance-related problems was no longer significant, indicating a complete mediation (see Fig. 4). Approximately one fourth of the total effect was explained by the indirect effect (P̂_M_ = 0.26; [71]).

**Fig. 5:**
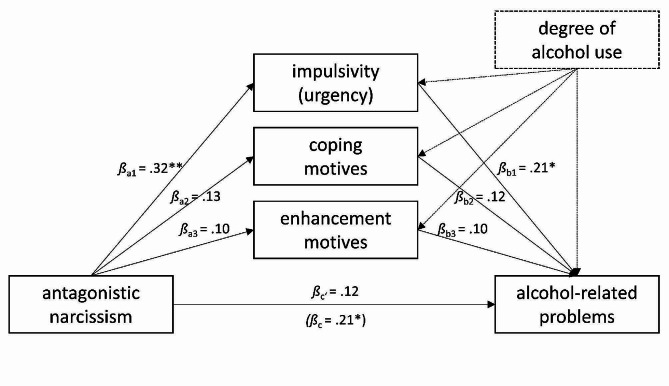
Parallel mediation model linking antagonistic narcissism to alcohol-related problems while controlling for the degree of alcohol use. *Note*: *N* = 98. Alcohol-related problems were assessed at T2, all other variables at T1. * *p* <.05, ** *p* <.01

**Mediators of the association between antagonistic narcissism and alcohol-related problems**. The regression of alcohol-related problems on antagonistic narcissism, controlling for the degree of alcohol use, was significant (*β*_c_ = 0.21, *p* =.029). Antagonistic narcissism significantly predicted impulsivity (*β*_a1_ = 0.32, *p* =.001), and impulsivity subsequently predicted alcohol-related problems (*β*_b1_ = 0.21, *p* =.043). In contrast, antagonistic narcissism did not significantly predict enhancement or coping motives and neither enhancement nor coping motives did subsequently predict alcohol-related problems (*p* >.05). The indirect effect through impulsivity was not significant (*β*_a1b1_ = 0.07, 95% CI [-0.002, 0.172]), although it had a similar effect size to the significant indirect effect through impulsivity in the model predicting total substance-related problems, indicating a lack of power rather than different mechanisms. Supporting this interpretation, approximately one third of the total effect was explained by the indirect effect of impulsivity (P̂_M_ = 0.32; [71]). After accounting for the indirect effects, the effect of antagonistic narcissism on alcohol-related problems was no longer significant, indicating a complete mediation (see Fig. 5).

**Fig. 6:**
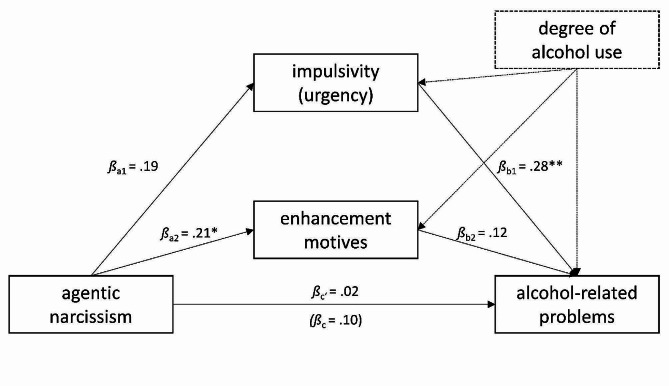
Parallel mediation model linking agentic narcissism to alcohol-related problems while controlling for the degree of alcohol use. *Note*: *N* = 98. Alcohol-related problems were assessed at T2, all other variables at T1. * *p* <.05, ** *p* <.01

**Mediators of the association between agentic narcissism and alcohol-related problems**. The regression of alcohol-related problems on agentic narcissism, controlling for the degree of alcohol use, was not significant (*β*_c_ = 0.10, *p* =.31). Again, note that this is not equivalent to the partial correlation reported in Sect. “Substance-related problems controlled for the degree of use”, as the mediation model relies on the subsample that provided SUMM data concerning alcohol and thus has less power. Agentic narcissism predicted impulsivity at trend level (*β*_a1_ = 0.19, *p* =.064), and impulsivity was subsequently related to alcohol-related problems (*β*_b1_ = 0.28, *p* =.003). In contrast, agentic narcissism significantly predicted enhancement motives (*β*_a2_ = 0.21, *p* =.039) but enhancement motives did not subsequently predict alcohol-related problems (*p* >.05). The individual indirect effects through impulsivity and enhancement motives were not significant, but the total indirect effect was significant (*β*_ab_ = 0.08, 95% CI [0.006, 0.179]), suggesting that impulsivity and enhancement motives may jointly explain the association between agentic narcissism and alcohol-related problems (see Fig. 6). The indirect effects through impulsivity and enhancement motives explained about half (P̂_M_ = 0.54) and one fifth (P̂_M_ = 0.17; [71]) of the total effect, respectively.

## Discussion

This preregistered study applied a dimensional approach to investigate associations of narcissism with substance use and substance-related problems as well as potential mediators of these associations. We found an association of narcissism with substance-related problems beyond the degree of substance use. This effect was even more pronounced predicting substance-related problems one year later, supporting the temporal unfolding of this effect. The prospective effect of narcissism on substance-related problems beyond the degree of use was reflected in uniquely grandiose aspects as well as aspects of narcissism common to grandiosity and vulnerability, but not in uniquely vulnerable aspects of narcissism. Associations of grandiose narcissism and antagonistic narcissism, the core constituent dimension of narcissism, with substance-related problems were fully mediated by impulsivity, but not mediated by substance use motives. Tentative evidence suggests that enhancement motives, related to the central goal of agentic-narcissistic self-regulation to maintain a grandiose self [53], may play a role alongside impulsivity in mediating the association of agentic narcissism and substance-related problems.

This is the first study to show that the association of narcissism with substance-related problems in (poly-)substance users is not attributable to an underlying association with the degree of substance use, highlighting narcissism as a putative specific risk factor for the development of SUDs. Our results show that individual differences in potentially relevant personality characteristics, such as antagonistic narcissism, may help identify which substance users might be at risk for the development of substance-related problems. We further provide first evidence on the putative mechanisms underlying the specific link of narcissism to substance-related problems, highlighting the role of impulsivity, which may inform future research and eventually enhance prevention and treatment.

### Narcissism and substance use

Against expectations, we found no evidence for associations between narcissism and the degree of substance use in a substance-using population. Though this finding seems to contradict previous studies reporting such associations in populations not specifically sampled to include only substance users (mostly college students; 17–21), we note that our sample comprised only individuals who actively used at least one substance. From this, it may be concluded that dimensions of narcissism do not predict the extent of substance use within those who went beyond a general “substance use threshold”. The differences to previous studies might also result, however, from the use of different measures. Specifically, previous studies partially used measures of pathological narcissism in terms of concurrent grandiosity and vulnerability [27] or the Narcissistic Personality Inventory, which primarily tackle grandiose narcissism but were not designed to measure its constituent dimensions [21, 72]. In contrast, our approach was to disentangle different dimensions underlying grandiosity and vulnerability on the basis of the Five-Factor Model (Weiss et al., 2019).

### Narcissism and substance-related problems

While we observed no associations between narcissism and substance use, we did observe associations between grandiose narcissism– including both antagonistic and agentic aspects– and substance-related problems. This underlines the importance of distinguishing between substance use and substance-related problems in SUD research [29]. When examining the specific prospective effect of grandiose narcissism on substance-related problems controlled for the degree of substance use, this association seemed to be primarily driven by the constituent factor antagonistic narcissism, the common core of both grandiose and vulnerable narcissism. Hence, in line with our hypotheses, a constituent dimension of vulnerable narcissism– antagonistic narcissism– was specifically related to substance-related problems beyond the degree of substance use, but, against expectations, this was not reflected in vulnerable but in grandiose narcissism.

We observed the same pattern when examining alcohol-related outcomes only. Supporting that antagonistic narcissism may contribute to substance-related problems across substances, antagonistic narcissism showed the strongest specific prospective effect on cannabis- and stimulant-related problems, although with slightly smaller effect size and not significant (potentially due to the smaller sample size in these subsamples). In contrast, for nicotine, we did not find any evidence for an effect of narcissistic traits on the development of nicotine-related problems beyond the degree of nicotine use. This may be due to the less severe psychoactive effects of nicotine as compared to other psychoactive substances, which may contribute to the different distribution of endorsed DSM-criteria in nicotine as compared to other substance use disorders [73]. This observation replicates in newer studies based on the DSM 5. In tobacco use disorder, symptoms grouped as social impairments are less frequent than in other SUDs (for example alcohol, [74, 75]), while craving and tolerance are more frequent. Narcissism may be more relevant for the symptoms that are more frequent in other SUDs than in nicotine use disorder.

Our results, highlighting antagonistic narcissism as the driving factor, may help to explain previous inconsistent findings regarding the role of vulnerable and grandiose narcissism for substance-related problems. Different studies have used different measures to assess grandiose versus vulnerable narcissism and the common constituent trait of antagonistic narcissism may have been reflected more in vulnerable narcissism in some and more in grandiose narcissism in other measures [6]. This may explain why some results implied vulnerable narcissism as more relevant while others indicated grandiose narcissism as more relevant [37, 43]. Only one other study has reported a specific association with substance-related problems (controlling for the degree of use) and this study also highlights grandiose narcissism as a predictor of substance-related problems [43], thus our results conform with prior evidence. This underlines the utility of the three-factor as compared to the two-factor solution of narcissism [11]. In terms of practical implications, among substance users, more narcissistic individuals may be at higher risk for substance-related problems although they do not show a higher degree of use. Preventive measures that sample at-risk individuals based on the degree of substance use might hence overlook narcissistic individuals who do not use as much as non-narcissistic counterparts with a similar risk to develop substance-related problems.

### Mediators of the association between narcissism and substance-related problems

The prospective specific effect of grandiose narcissism on substance-related problems was fully mediated by impulsivity, both across substances and in an alcohol-specific analysis, partly confirming our preregistered hypotheses. Against expectations, enhancement motives did not act as a mediator for the effect of grandiose narcissism on substance-related problems. In terms of the three factor model, this association of grandiose narcissism with substance-related problems was reflected both in antagonistic and in agentic narcissism. Examining the association of antagonistic narcissism with substance-related problems confirmed the role of impulsivity as a full mediator of this effect, mirroring what was observed for the superordinate factor grandiose narcissism. Examining the association of agentic narcissism, the uniquely grandiose constituent factor, with alcohol-related problems, revealed a somewhat different picture. Here, impulsivity and enhancement motives together, but not individually, mediated the specific prospective effect of agentic narcissism on alcohol-related problems. This is in line with the notion that enhancement motives may serve to achieve the agentic-narcissistic goal to maintain a grandiose self [53]. This mediating effect may only be uncovered using the more fine-grained three-factor model of narcissism, which can isolate uniquely grandiose aspects of narcissism in the agentic narcissism subscale [11]. Given that the specific prospective association between agentic narcissism and alcohol-related problems, which was significant in the full sample of alcohol users, was no longer significant in the smaller subsample that could be used for the mediation analyses (due to restraints on the SUMM data), these results and conclusions should be treated as preliminary. Future studies may furthermore use a different assessment instrument. The enhancement motives subscale of the SUMM, which upon closer examination mainly targets self-enhancing strategies to have fun, may not be the ideal assessment instrument for what is encompassed by enhancement motives in terms of agentic-narcissistic self-regulation (self-enhancing strategies to maintain a grandiose self; [53]).

Conceptually, these findings suggest that both the “impulsivity hypothesis” as well as the “self-regulation hypothesis” may play a role. However, we did not find evidence for the second part of the self-regulation hypothesis addressed in this study, namely that vulnerable narcissism may go along with coping motives to use substances which would subsequently facilitate the development of substance-related problems.

The association between antagonistic narcissism and substance-related problems further conforms with the idea that antagonistic patterns of experience and behavior can be placed on the *externalizing spectrum* in structural models of personality and psychopathology such as the Hierarchical Taxonomy of Psychopathology (HiTOP; [76]), which stands in close proximity to SUDs. Within the externalizing spectrum, the HiTOP further differentiates externalizing-antagonistic from externalizing-disinhibited patterns of experience of behavior [77], which points to the important role of disinhibition/impulsivity in substance-related problems. As part of our preregistered hypotheses, we investigated whether associations between narcissism and substance-related problems would be better explained by self-regulation or by impulsivity. Contrary to our hypothesis, that posited self-regulation, particularly through coping or enhancement motives, as a more crucial mediator than impulsivity, our findings revealed that impulsivity was in fact the most significant mediator across all models. For psychotherapeutic practice, this means that impulsivity might be a particularly valuable target for interventions aiming to reduce substance-related problems in individuals scoring high in grandiose aspects of narcissism.

### Limitations and future directions

First, the variation in sample sizes across different substances reduced the likelihood of detecting effects for some substances. Therefore, we refrain from interpreting the lack of significant effects in nicotine, cannabis and stimulant users as a lack of true effects and encourage future research to examine substance-specific effects based on larger samples. Second, substance-specific and SUMM subsamples had different sample sizes. In consequence, correlation coefficients and *p*-values differed between the subsamples of the same substance, and the subsamples used for the mediation analyses were selective such that they included only participants that deemed the respective substance relevant to them. Future studies should assess substance use motives for all substances of interest for the analyses and may include other measures to assess narcissistic self-regulation. Third, for the mediation analyses examining effects on total substance-related problems (across substances), the substance-related problems score was based on all measured substances, while the mediator variable was based on the two most relevant variables only. To enhance the validity of the total score of substance use motives, future studies should include all substances. Fourth, we have focused on two literature-based potential mechanisms of SUD in narcissism. However, other mechanisms, for example comorbid mental health conditions, or explanatory underlying factors, for example socioeconomic status, are plausible and may complement or even explain our findings. Future studies with larger samples are needed to rule out such alternative explanations. Last, although we used one year follow-up data for the dependent variable, this is not equivalent to longitudinal, causational mediation, which would require repeated measures at three measurement points. Longitudinal designs with three measurement points including repeated measures of all variables in the model are warranted for testing causational hypotheses.

### Conclusions

This preregistered, combined confirmatory and exploratory study establishes narcissism as a specific correlate of substance-related problems, implying SUD risk. We did not observe any associations between narcissism and the degree of substance use. Rather, our findings reveal a temporally stable link between narcissism and substance-related problems that is not explained by an underlying association with the degree of substance use but reflects a specific role for the development of SUD symptoms. This was driven primarily by antagonism, the common core of grandiose and vulnerable narcissism, and by agentic narcissism, the uniquely grandiose aspect of narcissism. Importantly, our results highlight the unique role of impulsivity as a mediator of the associations of grandiose narcissism and antagonism with substance-related problems. Additionally, the tentative identification of enhancement motives as a potential mediator for agentic narcissism underscores that self-regulation may play an additional role in SUD risk conveyed by uniquely grandiose aspects of narcissism. Similar patterns of results for total substance-related problems and alcohol-related problems indicate that these mechanisms may be similar across substances, except for nicotine. In conclusion, narcissistic individuals may not use substances to a higher degree, but for different reasons, which may facilitate the development of substance-related problems. To understand these fine-grained differences between dimensions of narcissism and related mechanisms may improve targeted prevention and treatment.

### Electronic supplementary material

Below is the link to the electronic supplementary material.

Supplementary Material 1

## Data Availability

The preregistration, code and dataset generated and/or analysed during the current study are available in the Open Science Framework repository, [https://osf.io/xrqbv/].

## Abbreviations

D-ISU: Dresden Inventory of Substance Use
FFNI-BF: Five Factor Narcissism Inventory– Brief Form
FFM: Five Factor Model (of personality)
HiTOP: Hierarchical Taxonomy Of Psychopathology
SCID-5 CV: Structured Clinical Interview for Psychological Disorders 5 Clinician Version
SUD: Substance Use Disorder
SUMM: Substance Use Motives Measure
UPPS-P: Urgency Perseverence Premeditation Sensation Seeking– Positive Urgency Impulsive Behavior Scale
