# Supplementary material for: Impulsivity mediates the association between narcissism and substance-related problems beyond the degree of substance use: a longitudinal observational study
Source: BMC Psychiatry. 2024 Apr 15;24:280. doi: 10.1186/s12888-024-05718-y (PMC11017556; doi:10.1186/s12888-024-05718-y)
Supplement: Supplementary file 1 — Supplementary Material 1 [file 12888_2024_5718_MOESM1_ESM.pdf]

Supplemental Methods

To facilitate the comparison between preregistered methods and what was actually carried out in this study, below, we quote literally the relevant sections of the preregistration and outline all changes (if any) behind each paragraph.

Hypotheses

“[We] hypothesize that the positive correlation between narcissism and the degree of substance use as well as substance-related problems from previous studies can be replicated. Regarding the underlying mechanisms, [we] expect that different substance use motives explain the association between narcissism and the degree of substance use as well as substance-related problems better than impulsivity […]. The preliminary hypotheses are as follows:

(I) Grandiose and vulnerable narcissism are associated with the degree of substance use.

(IIa) The association of the degree of substance use with vulnerable narcissism is mediated more strongly through coping motives than through impulsivity.

(IIb) The association of the degree of substance use with grandiose narcissism is mediated more strongly through enhancement motives than through impulsivity.

(III) Grandiose and vulnerable narcissism are associated with substance-related problems.

(IVa) The association of substance-related problems with vulnerable narcissism is mediated more strongly through coping motives than through impulsivity.

(IVb) The association of substance-related problems with grandiose narcissism is mediated more strongly through enhancement motives than through impulsivity.”

Changes referring to this section:

In the preregistration, we listed only the hypotheses positing differential effects of the mediators on the respective association, but we also test the implicit precursory conditions that both mediators are significant individually. We added the degree of substance use as a control variable to the mediation models prospectively predicting substance-related problems.

Questionnaire Measures

"To limit the number of comparisons while allowing substance-specific results, only the four most commonly used substances, namely alcohol, nicotine, cannabis and amphetamine as well as the total degree of substance use will be used for the analyses."

Changes referring to this section:

Rather than using only the data on amphetamines, we computed a stimulant score including other stimulants (e.g. cocaine) to increase sample size.

Data Analysis

"All analyses will be conducted in R. Variables with skewed distribution will be log-transformed. Mediation analyses will be carried out with the PROCESS macro assessing the parallel mediation model (model 4.2) in R.

To test hypothesis (I) a bivariate analysis of correlation between vulnerable as well as grandiose narcissism and the degree of substance use will be carried out for the four substances and the degree of substance use score. Only significant correlations will be further investigated in the analyses for hypothesis (II).

To test hypothesis (IIa) and (IIb), a logistic regression path analysis with the degree of current substance use as dependent variable, the independent variable vulnerable (IIa)/grandiose (IIb) narcissism and the two mediator variables (1) coping motives for substance use (IIa)/enhancement motives for substance use (IIb) and (2) impulsivity will be conducted for each substance and total degree of substance use score. The contrast parameter between indirect effect mediated by mediator 1 and indirect effect mediated by mediator 2 will be computed.

To test hypothesis (III), a bivariate analysis of correlation between vulnerable as well as grandiose narcissism and substance-related problems will be carried out for the four substances and the substance-related problems score. Only significant correlations will be further investigated in the analyses for hypothesis (IV).

To test hypothesis (IVa) and (IVb), a logistic regression path analysis with substance-related problems as dependent variable, the independent variable vulnerable (IVa)/grandiose (IVb) narcissism and the two mediator variables (1) coping motives for substance use (IVa)/enhancement motives for

substance use (IVb) and (2) impulsivity will be conducted for each substance and substance-related problems score. The contrast parameter between indirect effect mediated by mediator 1 and indirect effect mediated by mediator 2 will be computed..”

Changes referring to this section:

We used the ordered quantile normalization transformation due to better performance (Peterson & Cavanaugh, 2020).

Supplemental Results

Table S1

Self-reported ethnicity of participants at T1

Ethnicity	N	%
Caucasian	130	93.5
Asian	1	0.7
Near/middle east	1	0.7
Sinti and Roma	2	1.4
Native american	1	0.7
Other	4	2.8

Table S2

T-tests of mean differences in narcissistic traits between substance users vs. non-users by substance

**laboratory session**	nicotine use				cannabis use				stimulant use			
	M(SD)				M(SD)				M(SD)			
	yes	no	*t*	*d*	yes	no	*t*	*d*	yes	no	*t*	*d*
Grandiose N.	54.3 (11)	58.8 (11.1)	-1.80	-.41	55.7 (11.1)	52.2 (10.9)	1.45	.31	54.8 (11.5)	55.3 (10.7)	-0.25	-.04
Vulnerable N.	21.7 (5.2)	22.7 (5.9)	-0.78	-.18	22.1 (5.2)	21 (5.5)	0.99	.21	21.8 (5.7)	22 (4.8)	-0.2	-.03
Agentic N.	22.6 (6.3)	24.4 (5)	-1.29	-.3	23.2 (6.1)	21.4 (6.3)	1.38	.3	22.6 (6)	23.3 (6.4)	-0.7	-.12
Antagonistic N.	37.1 (7.9)	40.4 (6.9)	-1.91	-.44	38.2 (7.8)	35.4 (7.6)	1.69	.36	37.8 (8.2)	37.4 (7.2)	0.36	.06
Neurotic N.	18.4 (5.2)	18.2 (5.6)	0.2	.05	18.6 (5.3)	17.4 (5.2)	1.1	.24	18.4 (5.4)	18.4 (5.2)	0.05	.01
**follow-up**	nicotine use				cannabis use				stimulant use			
	M(SD)				M(SD)				M(SD)			
	yes	no	*t*	*d*	yes	no	*t*	*d*	yes	no	*t*	*d*
Grandiose N.	54.2 (11.1)	57.3 (11.9)	-1.19	-.28	55.8 (11.7)	52.2 (10)	1.58	.32	54.9 (11.2)	54.7 (11.5)	0.09	.02
Vulnerable N.	21.8 (5)	22 (6.3)	-0.17	-.04	21.6 (5.2)	22.3 (5.2)	-0.67	-.14	21.8 (5.4)	21.8 (4.9)	0.04	.01
Agentic N.	22.7 (6.2)	23.9 (5.7)	-0.81	-.19	23.3 (6.2)	22 (5.9)	1.05	.21	22.6 (6.1)	23.4 (6.2)	-0.76	-.14
Antagonistic N.	36.8 (7.8)	39.4 (7.4)	-1.49	-.34	37.9 (7.9)	35.7 (7.4)	1.36	.27	37.8 (8.2)	36.5 (7.2)	0.86	.16
Neurotic N.	18.5 (5.1)	18 (5.8)	0.4	.09	18.1 (5.3)	19 (5.2)	-0.8	-.16	18.3 (5.1)	18.5 (5.7)	-0.23	-.04

Note. Narcissism (N.) was assessed during the laboratory session, substance use during the laboratory session (upper panel) and follow-up (lower panel), respectively. M = mean, SD = standard deviation, t = t-value from independent samples t-test, *d* = cohen's *d.* The degrees of freedom are 137 for all cross-sectional tests and 121 for all longitudinal tests. * $p < .05$

Table S3

Correlations between substance use variables and facets of narcissism with confidence intervals

	Grandiose N.	Vulnerable N.	Agentic N.	Antagonistic N.	Neurotic N.
**Degree of use (laboratory session)**					
Total	.01<br>[-.16, .17]	.06<br>[-.11, .22]	-.07<br>[-.23, .10]	.09<br>[-.08, .25]	.04<br>[-.13, .20]
Alcohol	.03<br>[-.13, .20]	.07<br>[-.10, .24]	.09<br>[-.08, .26]	.00<br>[-.16, .17]	.10<br>[-.07, .26]
Nicotine	.07<br>[-.12, .25]	.14<br>[-.05, .31]	-.00<br>[-.18, .18]	.16<br>[-.02, .33]	.06<br>[-.13, .24]
Cannabis	.04<br>[-.15, .22]	-.07<br>[-.25, .12]	-.12<br>[-.30, .07]	.11<br>[-.08, .29]	-.14<br>[-.32, .04]
Stimulants	-.17<br>[-.37, .06]	.14<br>[-.09, .35]	-.13<br>[-.34, .09]	-.10<br>[-.31, .13]	.20<br>[-.03, .40]
**Degree of use (follow-up)**					
Total	-.03<br>[-.20, .15]	.08<br>[-.10, .25]	-.10<br>[-.27, .08]	.07<br>[-.11, .25]	.04<br>[-.14, .22]
Alcohol	.02<br>[-.15, .20]	.18<br>[-.00, .34]	.05<br>[-.13, .22]	.06<br>[-.12, .24]	.15<br>[-.02, .32]
Nicotine	-.01<br>[-.20, .19]	.15<br>[-.05, .34]	-.09<br>[-.28, .11]	.14<br>[-.06, .33]	.01<br>[-.19, .20]
Cannabis	-.12<br>[-.32, .09]	-.08<br>[-.28, .14]	-.20<br>[-.39, .01]	-.04<br>[-.25, .17]	-.10<br>[-.30, .11]
Stimulants	-.16<br>[-.38, .08]	.06<br>[-.17, .29]	-.07<br>[-.30, .16]	-.15<br>[-.37, .08]	.10<br>[-.13, .33]
**Substance-related problems**					
Total	.11<br>[-.06, .27]	.15<br>[-.01, .31]	.07<br>[-.10, .23]	.17*<br>[.00, .33]	.12<br>[-.05, .28]
Alcohol	.16<br>[-.01, .32]	.11<br>[-.06, .27]	.18*<br>[.01, .34]	.14<br>[-.03, .30]	.11<br>[-.06, .27]
Nicotine	.09<br>[-.09, .27]	.15<br>[-.03, .32]	.06<br>[-.13, .24]	.14<br>[-.05, .31]	.07<br>[-.12, .25]
Cannabis	.00<br>[-.18, .19]	.05<br>[-.14, .23]	-.02<br>[-.20, .17]	.04<br>[-.15, .22]	-.03<br>[-.21, .16]
Stimulants	-.02<br>[-.24, .20]	.19<br>[-.03, .39]	-.07<br>[-.29, .15]	.08<br>[-.14, .30]	.18<br>[-.04, .39]

	Grandiose N.	Vulnerable N.	Agentic N.	Antagonistic N.	Neurotic N.
**Substance-related problems (follow-up)**					
Total	.17	.15	.07	.25**	.04
	[-.01, .33]	[-.03, .32]	[-.11, .24]	[.07, .41]	[-.13, .22]
Alcohol	.19*	.22*	.18*	.25**	.11
	[.01, .36]	[.05, .39]	[.00, .35]	[.08, .41]	[-.07, .28]
Nicotine	.04	.16	-.01	.14	.04
	[-.16, .23]	[-.03, .35]	[-.21, .19]	[-.06, .32]	[-.16, .23]
Cannabis	.05	-.05	-.05	.11	-.13
	[-.16, .26]	[-.25, .16]	[-.26, .16]	[-.10, .31]	[-.33, .08]
Stimulants	-.05	.14	-.12	.04	.15
	[-.28, .19]	[-.10, .36]	[-.34, .11]	[-.19, .27]	[-.09, .37]
**Substance-related problems controlled for degree of use**					
Total	.14	.16	.16	.15	.12
	[-.02, .30]	[-.01, .31]	[-.01, .32]	[-.02, .31]	[-.04, .28]
Alcohol	.17*	.09	.16	.17	.07
	[.00, .32]	[-.08, .25]	[-.01, .31]	[.00, .33]	[-.10, .23]
Nicotine	.06	.08	.07	.05	.04
	[-.12, .24]	[-.10, .26]	[-.11, .25]	[-.14, .23]	[-.14, .22]
Cannabis	-.04	.15	.10	-.06	.11
	[-.22, .15]	[-.04, .32]	[-.08, .28]	[-.24, .13]	[-.07, .29]
Stimulants	.13	.13	.03	.20	.07
	[-.09, .34]	[-.09, .34]	[-.19, .25]	[-.02, .40]	[-.16, .28]
**Substance-related problems controlled for degree of use (follow-up)**					
Total	.24**	.13	.18	.27**	.02
	[.07, .40]	[-.05, .30]	[.00, .34]	[.09, .42]	[-.16, .20]
Alcohol	.21*	.16	.18*	.26**	.03
	[.03, .37]	[-.02, .33]	[.00, .35]	[.08, .42]	[-.14, .21]
Nicotine	.06	.09	.06	.06	.04
	[-.14, .25]	[-.11, .28]	[-.14, .25]	[-.13, .26]	[-.16, .23]
Cannabis	.18	.00	.10	.19	-.09
	[-.03, .37]	[-.21, .21]	[-.11, .30]	[-.02, .38]	[-.30, .12]
Stimulants	.08	.13	-.10	.20	.11
	[-.15, .31]	[-.10, .35]	[-.32, .14]	[-.03, .41]	[-.13, .33]

Note. Values in square brackets indicate the 95% confidence interval for each correlation. Correlations for total substance-related problems and total degree of use are based on the total sample (N = 139). Correlations with the degree of use and substance-related problems for alcohol, nicotine, cannabis and stimulants are based on the alcohol using subsample (n = 138), the nicotine using subsample (n = 116), the cannabis using subsample (n = 112), and the stimulant using subsample (n = 78), respectively.
* indicates $p < .05$. ** indicates $p < .01$.
